# The Great Imitator: A Case of Accidental Kratom Overdose

**DOI:** 10.7759/cureus.43144

**Published:** 2023-08-08

**Authors:** Shajeda Ahmed, Quoc Vinh Tran, Mary McLean

**Affiliations:** 1 Emergency Medicine, Ross University School of Medicine, Bridgetown, BRB; 2 Emergency Medicine, AdventHealth East Orlando, Orlando, USA

**Keywords:** kratom overdose, kratom toxicity, naloxone, narcan, opioid, substance abuse, kratom

## Abstract

Kratom (*Mitragyna speciosa*) is an herb that is sold over the counter in both pill and liquid forms. It contains opioid and stimulant properties and is used for relaxation as well as for weaning off opioid addictions. While a few adverse effects of kratom have been already reported, mainly with concerns around its toxicity, very little is known about it. We report a case of a female in her 40s presenting with signs of hypoxia reversed with naloxone administration, initially suspected to be a case of opioid overdose. Upon becoming alert and oriented, the patient and her husband reported that she consumed a large amount of kratom bought from the local gas station, and he later noticed that her lips were turning blue and she was becoming increasingly altered. Her urine toxicology was noted to be negative for opioids or any other substance use. The patient survived this accidental overdose due to the quick action of her husband, who rushed her to the emergency department (ED) upon realizing she appeared altered and very ill. It is important for emergency medicine practitioners to be aware of kratom overdose as a possible item on the differential diagnosis. This paper focuses on kratom overdose presentation and treatment.

## Introduction

Kratom (*Mitragyna speciosa*) is an herbal substance that is native to Southeast Asia and has both opioid-like and stimulant properties. It is consumed orally by chewing the leaves, ingesting the powdered leaf in a pill or capsule form, smoking it, or drinking it with the powdered leaf dissolved in a beverage [1]. The average amount of the active compound (mitragynine) in one serving of “kratom juice” is 79 milligrams, and the average daily intake recommendation from brands is 276.5 milligrams [2]. Recently, it has become popular in the United States and is available over the counter [3]. At low doses (1-5 grams), kratom acts like a stimulant with the effects of increased alertness, social behavior, and physical energy. At high doses (5-15 grams), it brings on euphoria and has opioid-like properties such as decreased pain stimulation [4].

While some adverse effects of this herb have been reported, the data is limited to case reports. Adverse effects include respiratory depression, seizures, and opioid-like withdrawal symptoms when a user stops kratom use abruptly [5]. The Drug Enforcement Administration lists kratom as a Drug and Chemical of Concern [6]. There is limited literature on kratom toxicity and its clinical presentation, and thus, it is easily missed in the emergency department (ED) differential diagnosis. Due to its opioid-like properties, patients who overdose on kratom do respond to naloxone administration [7].

This case involves a patient presenting with unresponsiveness, apnea, cyanosis, and constricted pupils raising suspicion of opioid overdose and prompting immediate naloxone administration, which (along with supplemental oxygen and bag valve mask) resolved her symptoms. However, urine toxicology results were negative, and it was later revealed that high-dose kratom had recently been ingested. The patient’s symptoms returned during an observation period in the ED. The Poison Control Center advised that kratom overdose can cause rebound hypoxia 12-24 hours after ingestion. It is crucial for clinicians to be aware of rebound hypoxia when deciding on safe disposition for patients from the ED. Specifically, patients must be observed for this 24-hour high-risk time period, and if the patient leaves against medical advice (AMA) with a companion despite this medical recommendation, they should be given naloxone with clear instructions in anticipation of rebound symptoms and hypoxia. By highlighting this case, our goal is to raise awareness among ED practitioners regarding kratom toxicity masquerading as opioid overdose and the high risk for rebound hypoxia even if symptoms completely resolve after initial naloxone administration.

## Case presentation

A female in her 40s with a past medical history of depression and anxiety was brought to the ED by her husband in a private vehicle, presenting with altered mental status. On arrival, she appeared apneic and unresponsive. Her pulse oxygenation was initially unmeasurable, but her lips and extremities appeared cyanotic. Her pupils were initially 1 millimeter in diameter. She was immediately placed on a bag valve mask with supplemental oxygen. Her score on the Glasgow Coma Scale was 3, and point-of-care glucose was 203 milligrams/deciliter. After bagging with supplemental oxygen, her pulse oxygenation was noted to be 90%. While the team was getting IV access and giving naloxone, the airway cart was being set up, and a respiratory therapist was called to the ED.

Due to the patient’s presentation of pinpoint pupils, altered mental status, hypoxia, and respiratory depression, naloxone was given per the ER protocol. Shortly after 2 milligrams of intravenous naloxone administration, the patient became more responsive and alert, and her pupil diameter increased to 5 millimeters. Given her responsiveness to naloxone, opioid overdose was suspected. However, as soon as the patient was more alert and able to converse, she adamantly denied opioid use.

At this point, the patient’s husband reported that he noticed her lips turning blue earlier that morning, after which time she quickly became unresponsive, prompting him to bring her to the hospital. He asserted that she did not consume any opiate substances. After further questioning, the patient herself noted that she did try over-the-counter kratom from the gas station for the first time that morning, eight capsules (400 milligrams/capsule) and a full bottle of liquid kratom extract (three times the normal strength). She reported taking the kratom to manage anxiety symptoms. With some time on the nasal cannula, the patient’s oxygen saturation improved to 100%.

Laboratory work including complete blood count (CBC), comprehensive metabolic panel (CMP), and venous blood gas (VBG) were ordered, and the results are shown in Table 1. Additionally, creatine kinase, magnesium, acetaminophen level, salicylate level, ethanol level, and urine toxicology were negative or normal. Chest X-ray showed only mild bibasilar atelectasis, computed tomography of the head showed no acute intracranial or other abnormalities, and the electrocardiogram showed sinus tachycardia with no ischemic-appearing ST segment elevations or depressions or other abnormalities (the corrected QT interval was 406 milliseconds, and all other intervals were normal).

**Table 1:** Laboratory work including CBC, CMP, and VBG CBC: complete blood count, CMP: comprehensive metabolic panel, VBG: venous blood gas, WBC: white blood cells, RBC: red blood cells, Hgb: hemoglobin, HCT: hematocrit, MCV: mean corpuscular volume, MCH: mean corpuscular hemoglobin, MCHC: mean corpuscular hemoglobin concentration, RDW: red blood cell distribution width, PLT: platelet, MPV: mean platelet volume, Na: sodium, K: potassium, Cl: chloride, CO_2_: carbon dioxide, BUN: blood urea nitrogen, ALT: alanine transaminase, AST: aspartate aminotransferase, Alk phos: alkaline phosphatase, A/G ratio: albumin/globulin ratio, eGFR: estimated glomerular filtration rate, pCO_2_: partial pressure of carbon dioxide, pO_2_: partial pressure of oxygen, HCO_3_: bicarbonate, SO_2_: oxygen saturation

Panel/laboratory work	Value	Reference ranges	Units
CBC			
WBC	13.94	3.8-10.4	10^3^/uL
RBC	4.62	4.2-5.7	10^3^/uL
Hgb	13.3	11.9-14.8	g/dL
HCT	40.8	35-43	%
MCV	88.3	82.5-98	fL
MCH	28.8	25-35	pg
MCHC	32.6	32.5-35.2	g/dL
RDW	13.6	11.4-13.5	%
PLT	379	150-450	10^3^/uL
MPV	9.8	8-12	fL
CMP			
Na	141	136-146	mmol/L
K	4	3.5-5	mmol/L
Cl	104	95-105	mmol/L
CO_2_	24	22-28	mmol/L
Anion gap	13	-	mmol/L
BUN	17	7-18	mg/dL
Creatinine	0.79	0.6-1.2	mg/dL
Glucose	224	<140	mg/dL
Calcium	9.2	8.4-10.2	mg/dL
ALT	15	10-40	U/L
AST	12	12-38	U/L
Alk phos	71	25-100	U/L
Protein (total)	7.4	6.0-7.8	g/dL
Albumin	4.70	3.5-5.5	g/dL
Globulin	2.7	2.3-3.5	g/dL
A/G ratio	1.7	1-2.5	-
Bilirubin (total)	<0.20	0.1-1	mg/dL
eGFR	96.5	-	mL/minute/m^2^
VBG			
pH	7.300	7.35-7.45	-
pCO_2_	58.8	35-45	mmHg
pO_2_	61.6	75-100	mmol/L
HCO_3_	29	22-28	mmol/L
SO_2_	87.9	94-100	mmol/L

After a short period of ED observation, during which the patient was on a nasal cannula, the patient was transitioned to room air, at which point she was noted to have an oxygen saturation of 99%. After a short period of time, her oxygen saturation was noted to drop again to 92%, and her pupil size decreased back to 2 millimeters. Per a conversation with Poison Control, patients who overdose on kratom are at risk for rebound respiratory depression within 12-24 hours. They recommended supportive care, additional naloxone if required, and 24 hours of observation.

The patient was placed back on supplemental oxygen at 3 liters per minute via nasal cannula. Her respiratory rate was 12 breaths/minute, and she was still easily arousable, so additional naloxone was not administered. However, given the recommendations from Poison Control, the decision was made to admit the patient for observation. However, the patient chose to leave AMA due to personal obligations at home. As she was hemodynamically stable at that moment, was able to tolerate oral liquids and solids, was alert, oriented, and speaking cohesively, had decision-making capacity, and had her husband to care for her, there was no criteria to hold the patient involuntarily. She agreed to follow up with her primary care practitioner and return to the ED if any new or worsening symptoms occurred. She was signed out AMA with her husband but was given a naloxone kit in the department, and both the patient and her husband were instructed on how to use it if necessary.

In this case of a female in her 40s brought in by her husband appearing hypoxic, obtunded, difficult to arouse, and cyanotic, our differentials included substance use disorder (opioid versus benzodiazepines versus other substances that could cause respiratory suppression), stroke, respiratory failure secondary to infection, cardiogenic causes, and central nervous system infections. Our workup included CBC, BMP, blood gas, DI-60, magnesium, salicylate level, acetaminophen level, urine drug screen, non-contrast computed tomography of the head, chest X-ray, and electrocardiogram to rule out some of these differential items. Due to the patient’s responsiveness to naloxone, high on our differential was opioid use disorder with overdose. However, to our surprise, the patient’s urine toxicology was negative for opioid or fentanyl use. Fortunately, in our case, the patient became alert shortly after interventions and was able to give us a history of large-volume kratom consumption.

## Discussion

A review of the literature demonstrated that *Mitragyna speciosa* (kratom) is an opioid receptor agonist, activating the mu and delta receptors. In the United States, it is becoming increasingly popular, especially by patients for self-treatment of opioid withdrawal [8]. Kratom works by acting as a stimulant at low doses, but at higher doses, it can bring on euphoria and act similarly to sedatives by reducing pain stimulation. At low doses (1-5 grams), kratom increases alertness, talkativeness, social behavior, and physical energy but causes loss of muscle coordination. At high doses (5-15 grams), kratom causes tachycardia, constipation, dizziness, hypotension, dry mouth, and sweating [4]. Perhaps, this agonistic-antagonistic relationship is a possible explanation for the rebound respiratory depression experienced by patients who overdose [9]. While there are kratom brand-based recommendations for dosing, the potency of kratom-containing products (e.g., capsules, tablets, powders, teas, liquid shots, and liquid extracts) is widely variable and may lead to an increased risk of overdose, particularly when a patient is trying a certain product for the first time.

Kratom withdrawal symptoms include muscle spasms, pain, insomnia, rhinorrhea, fever, appetite suppression, diarrhea, restlessness, tension, anger, and nervousness. The average amount of the active compound (mitragynine) in one drink of kratom is 79 milligrams. It has been noted that users who drink more than three glasses of kratom per day have a higher likelihood of developing dependence and withdrawal symptoms [2]. Aside from case reports, currently, there are no active clinical trials supporting these findings. As such, many ED practitioners are unaware that kratom toxicity is an increasing issue or that it can present similarly to opioid toxicity.

Opioid overdose presents with altered mental status, decreased respiratory drive, rate, and tidal volume, constricted pupils, and decreased bowel sounds [7]. The best predictor of respiratory toxicity is a respiratory rate of less than 12 breaths/minute [7]. Intravenous naloxone is given to patients suspected of opioid overdose. Naloxone serves as an antidote, and with adequate dosing, it will ensure that the patient is properly ventilated. Generally, if there are no signs of opioid withdrawal, there is no current maximum safe dose of naloxone. However, if the patient does not clinically improve after 5-10 milligrams, the diagnosis of opioid withdrawal should be reconsidered [7].

This case report is significant because kratom toxicity can be easily overlooked when it is misunderstood or unheard of. ED practitioners are vigilant for opioid overdose and are likely to give a single dose of naloxone to the undifferentiated patients presenting with these symptoms. However, if urine toxicology is negative for opioids and fentanyl, they may opt not to give further naloxone for continued symptoms. Clinicians should be cautious with kratom overdose, as this is associated with rebound hypoxia within 24 hours, which must be accounted for during patient management. A thorough toxicology workup is crucial to rule out other causes of hypoxia and substance ingestion, but there is currently no urine or serum test that detects kratom ingestion. Per the Florida Poison Control Center, kratom is associated with a risk of rebound hypoxia within 24 hours. An extensive literature search demonstrated that little is currently known about kratom toxicity management, and there are no current articles discussing rebound hypoxia from kratom.

As such, it is crucial for ED practitioners to understand this and to ensure patients are admitted for observation in cases of severe toxicity requiring naloxone, even if clinical symptoms appear promising in the first few hours after presentation. In cases of leaving AMA with full decision-making capacity, the patient and companions should be given naloxone and instructions upon signing out of the department.

## Conclusions

This case highlights the importance of recognizing kratom overdose, which can mimic opioid toxicity. Unlike opioid toxicity, kratom toxicity presents a continued risk for rebound symptoms and hypoxia 12-24 hours after the initial ingestion. In this case, we were fortunate that our patient could eventually self-report high-dose kratom consumption and that a urine sample could be collected to show toxicology studies negative for opioids and fentanyl, further alerting practitioners that this was not just an ordinary opioid overdose. This prompted the medical recommendation for 24-hour observation, and when the patient decided to leave AMA, it prompted naloxone kit distribution, explicit instructions on naloxone use, and a warning that rebound symptoms and hypoxia were a risk. For many other patients, misdiagnosis of kratom overdose as an opioid overdose could result in failure to recognize the risk of delayed rebound symptoms and hypoxia. ED practitioners must be aware of the rising popularity of kratom, must be vigilant for kratom toxicity in naloxone-responsive overdose with negative urine toxicology, and should recommend 24-hour observation to avoid harm from rebound hypoxia.
